# A Rare Case of Varicella-Zoster Virus Meningitis With High Intracranial Pressure in an Immunocompetent Adult

**DOI:** 10.7759/cureus.52437

**Published:** 2024-01-17

**Authors:** Awad Al Harbi, Jameelah Saeedi, Enas Almowalad, Razan Alahmari, Hana A Alzuabi, Leenah Almanea, AlAnoud AlAbdulhadi

**Affiliations:** 1 Neurosciences, King Abdullah Bin Abdulaziz University Hospital, Princess Nourah Bint Abdulrahman University, Riyadh, SAU; 2 Neurology, King Abdullah Bin Abdulaziz University Hospital, Riyadh, SAU; 3 College of Medicine, King Abdullah Bin Abdulaziz University Hospital, Princess Nourah Bint Abdulrahman University, Riyadh, SAU; 4 Medicine and Surgery, Princess Nourah Bint Abdulrahman University, Riyadh, SAU; 5 College of Medicine, Princess Nourah Bint Abdulrahman University, Riyadh, SAU; 6 College of Medicine, Alfaisal University, Riyadh, SAU

**Keywords:** vzv meningitis, high icp, meningitis, vzv, immunocompetent, varicella-zoster virus

## Abstract

Varicella-zoster virus (VZV) is an alphaherpesvirus causing varicella (chickenpox) and herpes zoster. While varicella typically presents with a vesicular rash, latent VZV may reactivate within the sensory ganglia causing shingles, characterized by painful vesicular rash, which may lead to neurologic complications such as aseptic meningitis. This case explores an atypical presentation in an immunocompetent young man with VZV meningitis lacking the characteristic skin rash but featuring elevated intracranial pressure. A literature review revealed rare instances of VZV-related neurologic disease without typical skin manifestations, suggesting the virus’s potential to affect the central nervous system directly. Treatment with intravenous acyclovir is recommended, with ganciclovir as an alternative treatment option. This case emphasizes the importance of considering VZV meningitis in the differential diagnosis of patients presenting with viral meningitis symptoms, with or without dermatomal rash or immunocompromised conditions.

## Introduction

Varicella-zoster virus (VZV) infection is a primary infection with VZV which results in varicella chickenpox described as vesicular lesions and shingles caused by the reactivation of latent VZV infection within the sensory ganglia. VZV typically shows painful vesicular rash [1], but can lead to neurological complications such as aseptic meningitis either with or without skin manifestation. Most cases are seen in immunocompromised patients, and only a few cases have been reported in the literature in immunocompetent patients [2]. One of the rare presentations secondary to viral meningitis is idiopathic intracranial hypertension (IIH) [3]. Here, we present an immunocompetent young male who presented with VZV meningitis associated with high IIH.

## Case presentation

A 38-year-old male contractor, with a known case of gout on allopurinol, presented to the emergency department (ED) with a history of headache and fever. He developed a severe headache four days before his visit. It started gradually, localized to the occipital region, and then involved all head regions. The headache was associated with photophobia, nausea, occasional vomiting, and poor appetite. On ED presentation, his temperature reached 38°C, which improved with paracetamol. The patient denied any history of similar headaches or recent travel. There was no history of mental status changes, focal neurological deficits, changes in vision, skin rash, seizures, or aura.

He had undergone dental procedures and had a dental abscess about 10 days before the headache, which was treated with antibiotics for two weeks. He also complained of pain in the hips, neck, and back.

His past medical and surgical history was unremarkable. There is no family history of similar presentation or neurological diseases. He was fully vaccinated with no childhood history of chickenpox. He had a history of IIH in 2003 and was admitted and treated at another institution for six to eight months. Cerebrospinal fluid (CSF) at that time showed high CSF pressure. No medical records were available for detailed information.

On examination, he was alert, oriented, and fluent in speech. Fundoscopic examination showed blurred lateral disc margins, visual acuity was 20/20 in both eyes, full extraocular movement bilaterally, no nystagmus, normal facial sensation and expressions, and normal motor and sensory examination in all extremities. Power was 5/5 in all extremities. There was no neck stiffness, but he complained of pain when bending the neck. The patient had symmetrical intact deep tendon reflexes and plantar flexion. The cerebellar examination was intact. He had a normal gait. No skin rash or lesions were appreciated. Chest, cardiovascular, and abdominal examinations were unremarkable. Upon neuro-ophthalmology evaluation, he was found to have grade 1-2 disc swelling in both eyes associated with some changes on the inferior arcuate; OD was -15 with non-specific changes and the superior arcuate; OS was -21.7 with clover-leaf peripheral visual field defect.

Investigations

Laboratory tests showed a white blood cell count of 10.80 × 10^3^/µL (4.0-11.0 × 10^9^/µL), neutrophil of 74.6% (49.0-81.0%), lymphocyte of 18.2% (14.0-41.0%), and hematocrit of 0.516 L/L (0.420-0.540 L/L). Blood cultures were negative. Comprehensive metabolic panel results included glucose and liver function tests. Renal function test, urine analysis, thyroid function test, C-reactive protein, troponin, electrolytes, activated partial thromboplastin time, and prothrombin time were within normal limits. Computed tomography of the head and chest radiograph were normal. A lumbar puncture showed an opening pressure of 35 mmHg.

CSF was clear with glucose of 3.2 mmol/L (2.2-3.9 mmol/L), elevated protein of 1.56 g/L (0.15-0.45 g/L), red blood cell count of 100 × 10^6^/L, and white blood cell count of 238 × 10^6^/L with 79% lymphocytes and 11% monocytes (Table 1). A meningitis multiplex polymerase chain reaction (PCR)-CSF panel was obtained for analysis which was positive for VZV and negative for herpes simplex virus I and II, human herpesvirus-6, parechovirus, cytomegalovirus, adenovirus, *Escherichia coli* K1 strain, *Haemophilus influenzae*, *Listeria monocytogenes*, *Neisseria meningitidis*, *Streptococcus agalactiae*, *Streptococcus pneumoniae*, parainfluenza, metapneumovirus, *Cryptococcus neoformans*, *Cryptococcus gattii*, and enterovirus. Additionally, human immunodeficiency virus testing, *Brucella* antibodies, and antinuclear antibodies were undetectable (Table 2).

**Table 1:** Initial CSF findings. CSF = cerebrospinal fluid

CSF characteristics	Result
CSF color	No color
Appearance spun	Colorless
CSF monocytes/macrophages (15–45%)	11%
CSF appearance	Clear
White blood cell count	238 × 10^6^/L
CSF lymphocytes (40–80%)	79%
RBC count spinal fluid	100 × 10^6^/L
Protein, CSF (0.15–0.45 g/L)	1.56
Glucose, CSF (2.2–3.9 mmol/L)	3.2

**Table 2:** Meningitis multiplex CSF-PCR results. CSF = cerebrospinal fluid; PCR = polymerase chain reaction

CSF-PCR interpretation	Result
VZV DNA PCR	Positive
Herpes simplex virus 1	Negative
Herpes simplex virus 2	Negative
*Escherichia coli* K1	Negative
Streptococcus agalactiae	Negative
Streptococcus pneumoniae	Negative
Cytomegalovirus	Negative
Haemophilus influenza	Negative
Listeria monocytogenes	Negative
Neisseria meningitidis	Negative
Human herpesvirus 6	Negative
*Cryptococcus neoformans*/*gattii*	Negative
Lyme PCR	Negative

Management

The patient was treated with intravenous (IV) acyclovir 10 mg/kg every eight hours for 10 days and ceftriaxone 2 g for 14 days. Moreover, he was started on acetazolamide 250 mg for one month and celecoxib 200 mg for pain management. Upon follow-up, he had improved significantly and reported complete recovery.

Follow-up

The patient had significant improvement and complete resolution of symptoms. The acetazolamide dose was tapered from 500 mg BID to 250 mg. He denied any side effects from the medication. During his follow-up appointment at the neuro-ophthalmology clinic, there was a noticeable improvement, especially in the visual field.

## Discussion

VZV is an alphaherpesvirus that causes varicella (chickenpox) and herpes zoster. Varicella typically causes mild-to-severe disease in children or immunocompetent patients with a disseminated vesicular rash. VZV remains latent in sensory cranial nerve ganglia or dorsal root ganglia after primary infection [4]. Reactivation of the VZV results in several neurologic complications, which include acute retinal necrosis, herpes zoster ophthalmicus, and aseptic meningitis. Advanced age and immunocompromised states are thought to be key risk factors for the development of herpes zoster and other VZV reactivation symptoms. A dermatomal rash and neuritis are the most prevalent symptoms [5].

Aseptic meningitis is a term that refers to any cause of inflammation of the brain meninges in which a CSF culture reveals no evidence of bacterial infection. It is characterized by symptoms such as headache, photophobia, nausea, and vomiting. VZV has been identified as the etiology of viral meningitis in 8-13% of cases. Meningitis is a rare consequence of VZV, occurring in only 0.5% of recorded cases [6]. VZV meningitis is uncommon in young and healthy individuals, with some cases presenting without the usual herpes zoster rash [6]. VZV is clinically identified by the occurrence of a dermatomal rash. It can also be diagnosed in the laboratory by evaluating vesicular skin material and CSF using PCR [1].

In our case, the patient presented with an atypical presentation of meningitis in an immunocompetent state. Although he manifested typical meningitis symptoms, he did not develop any skin rash related to VZV. Moreover, he had high intracranial pressure, which has been reported as a primary clinical characteristic of VZV meningitis in previous reports [7,8]. In this case, we cannot be certain if this was directly caused by meningitis or if he still had IIH from his previous medical history.

A thorough literature review revealed that VZV-related neurologic disease can occur even in patients with immune incompetence without typical herpes zoster exanthema. Numerous cases of VZV reactivation have been documented; six cases of VZV meningitis confirmed by PCR were reported but without any skin manifestations [9]. Additionally, Klein et al. reported the case of a 14-year-old patient with the same clinical appearance but no symptoms or indicators of elevated intracranial pressure [7]. Another case reported in 2018 by Ibrahim et al. discussed the case of a 15-year-old with predominant clinical features of increased intracranial pressure without a skin rash [8]. This would attest to the virus’s potential to spread directly from its latent state in the spinal ganglia to the brain and spinal cord without first affecting the skin.

The Infectious Diseases Society of America recommends IV acyclovir for the treatment of VZV meningoencephalitis. Ganciclovir may be used as an alternative agent [10]. In our case, the patient received IV acyclovir for 10 days with good response and improvement.

This case is being reported to keep VZV meningitis in the differential diagnosis for patients exhibiting clinical symptoms and CSF results consistent with viral meningitis in the absence of any dermatomal rash or immunocompromising medical conditions.

## Conclusions

This case indicates that, even in situations of aseptic meningitis in immunocompetent individuals without skin rash or other typical manifestations, it is reasonable to consider VZV in the differential diagnosis as it can raise intracranial pressure that can lead to symptoms and irreversible neurological disabilities which can be reversed with appropriate treatment.

## References

[1] Gershon AA, Breuer J, Cohen JI (2015). Varicella zoster virus infection. Nat Rev Dis Primers.

[2] Bhandari S, Alme C, Siller A Jr, Jha P (2021). Varicella zoster meningitis in immunocompetent hosts: a case series and review of the literature. WMJ.

[3] Kiefer L, Adam D, Mudugal D, Burnett MS (2020). Viral meningitis mimicking benign intracranial hypertension: a report of two cases. Interdiscip Neurosurg.

[4] Granerod J, Ambrose HE, Davies NW (2010). Causes of encephalitis and differences in their clinical presentations in England: a multicentre, population-based prospective study. Lancet Infect Dis.

[5] Nagel MA, Gilden D (2013). Complications of varicella zoster virus reactivation. Curr Treat Options Neurol.

[6] Fadhel M, Campbell N, Patel S, Mushtaq A, Fune L, Asif A, Hossain MA (2019). Varicella zoster meningitis with hypoglycorrhachia on cerebrospinal fluid (CSF) analysis in a young immunocompetent host without a rash. Am J Case Rep.

[7] Klein NC, McDermott B, Cunha BA (2010). Varicella-zoster virus meningoencephalitis in an immunocompetent patient without a rash. Scand J Infect Dis.

[8] Ibrahim W, Elzouki AN, Husain A, Osman L (2015). Varicella zoster aseptic meningitis: report of an atypical case and literature review. Am J Case Rep.

[9] Echevarría JM, Casas I, Tenorio A, de Ory F, Martínez-Martín P (1994). Detection of varicella-zoster virus-specific DNA sequences in cerebrospinal fluid from patients with acute aseptic meningitis and no cutaneous lesions. J Med Virol.

[10] Tunkel AR, Glaser CA, Bloch KC (2008). The management of encephalitis: clinical practice guidelines by the Infectious Diseases Society of America. Clin Infect Dis.

